# Exploiting contextual information to improve call prediction

**DOI:** 10.1371/journal.pone.0223780

**Published:** 2019-10-23

**Authors:** Mehk Fatima, Aimal Rextin, Shamaila Hayat, Mehwish Nasim

**Affiliations:** 1 Department of Computer Science & Information Technology, University of Lahore, Gujrat Campus, Gujrat, Pakistan; 2 Department of Computer Science, COMSATS University Islamabad, Islamabad, Pakistan; 3 Data61, CSIRO, Adelaide, Australia; College of EME, NUST, PAKISTAN

## Abstract

With the increase in contact list size of mobile phone users, the management and retrieval of contacts has becomes a tedious job. In this study, we analysed some important dimensions that can effectively contribute in predicting which contact a user is going to call at time *t*. We improved a state of the art algorithm, that uses frequency and recency by adding temporal information as an additional dimension for predicting future calls. The proposed algorithm performs better in overall analysis, but more significantly there was an improvement in the prediction of top contacts of a user as compared to the base algorithm.

## Introduction

Over the last decade, mobile phones have become ubiquitous and they provide a myriad of services to their users. The number of mobile phone users has increased from 12% in the year 2000 to more than 90% of the world’s population in 2014 [1]. Of late, smartphones are being used for: web browsing, video streaming, email etc., albeit making a call is still a very important function on a mobile phone. The contact list size on a smartphone has increased to an average of 308 contacts per phone [2] making the task of finding the desired contact more tedious. As a result, industry researchers, as well as end users, have started to explore different alternatives to make this task easier.

A typical smartphone user has two main ways through which he/she initiates a call: the contact book and the call log [3]. The contact list is a static list and the user needs to first search for the desired contact before the call initialization. This many user find inconvenient as are generally more likely to call a contact that they communicated with in the recent past. Hence many users prefer using the call log in which the call history is recorded in descending order starting from the most recent and going backward in time. A number of studies investigated how one can make it easier for the user to find the desired contact. In one such study, a new interface for the contact book was suggested in which the unused and less used contacts are displayed at the bottom in a small font [4], allowing the user to focus on the a subset of contacts that he/she generally contacts. However, many research studies investigated how a smaller list of 5–10 contacts can be predicted such that the desired contact is among this short list [2] [5] [6] [7]. The input for these prediction algorithms is the current time *t* and the call history of the user. This paper focuses on improving a recent call prediction algorithm proposed by Stefanis et. al [6] [8].

The two most widely used indicators for the likelihood of a user making a call to a contact is its frequency and recency. We note here that *recency* is a numerical score indicating how recently two users have communicated with each other, similarly, *frequency* is a score indicating how frequently the two users communicate [4]. One of the most used algorithms for predicting calls is the *Frequency-Recency (FR) Algorithm* that uses a linear combination of frequency and recency of contacts to calculate the likelihood of an user contacting a contact [6] [8]. Another recent work showed that mobile phone users also exhibit time-based periodic patterns in their communication [9]. These temporal features can be used to design several call prediction algorithms [3]. Inspired by these results, we plan to enhance the performance of the FR algorithm [8] by temporal patterns (periodicity). In this paper, we used a mix of qualitative and quantitative analysis.

### Contributions

The main contributions of this study, are listed below:

1. Research [3] has shown that there are periodic patterns in the calling behaviour of users. Inspired by this result, we showed that there is a peak hour in which a user-contact pair is likely to contact each other. We attribute this finding to *socio-temporal* pattern as discussed later in the paper.
2. We enhanced the FR algorithm [6] [8] which resulted in not only improving the accuracy of the FR algorithm, but it also resulted in fewer false negatives regarding the top contacts.

### Datasets

In this paper we will propose an improved algorithm and compare it with the FR algorithm. We will evaluate on two datasets: The smartphone dataset https://doi.org/10.5061/dryad.xpnvx0k9t [3] with 786 users collected in a developing country and the Nokia MDC dataset collected in Switzerland with 185 users [10]. The MDC dataset is collected by Nokia and details on how to get permission from Nokia to access the data is available online at https://www.idiap.ch/dataset/mdc. We removed all those participant’s data who had less than 100 calls. As a result, we were left with 522 users in the smartphone data set and 129 users in the MDC dataset respectively.

### Organization

The paper is organised as follows: Section summarizes some studies that relate to this paper. In Section, we present limitations of the FR algorithm [6] [8]. Section, presents a modified algorithm to overcome these limitations and we evaluate the effectiveness of this proposed algorithm in Section. Finally, in Section we will give our concluding remarks.

## Related work

Smartphones log a plethora of data, such as location, call history, application logs, etc. Such data is of immense use for the researchers to address various social, behavioural and economic issues. Smartphones have also been used as a tool in psychological research [11], for example, such data has been used to study personality traits, human mobility and activity patterns, trajectory estimation, human behaviour, and emotions in different situations [12] [13] [14] [15] [16] [17] [18]. Similarly, Call Detail Records (CDRs), have also been used to detect dense areas [19], to infer possible mode of transportation [20], to optimize public transport in a city [21], to deduce friendship network [22], to investigate the communication behaviour and demographics of mobile phone users [23].

Mobile phone call logs have been used to analyse calling patterns of users in order to improve call interfaces. Several studies, including [2] [5] [6] [7] have shown that contact book size is continuously growing, hence, making personal information management and retrieval a difficult task. Bentley et al. [2] found that the average size of the contact list has grown up to 308 contacts while in a previous study this size was 92 [5]. Bentley et al. [2] also found that more than 90% of communication occur with just the top 10 contacts. Bergman et al. [5] and Komninos et al. [4] supported these findings by showing that almost 47% contacts in modern smartphones have not been contacted for at least 6 months or have never been contacted at all.

These findings motivated researchers to try to come up with techniques to aid a user in finding a relevant contact when she intends to make a call. These techniques can be broadly classified into the following approaches:

**Design Based Approach:** In this approach, the manner in which contact list is displayed is changed. In general, contact lists are displayed in alphabetical order, but the continuous increase in the contact list size results in slower access to the desired contact. Hence, design-based approach changes the traditional display of the contact list in such a way that information retrieval task becomes quicker and easier. An example of this approach is the contact list proposed by Bergman et al. [5], which demotes rarely used contacts by placing them at the bottom of the screen and in a smaller font.
**Call Prediction Based Approach:** Another approach is to predict which contact a user is going to call, given some temporal and contextual data. It then computes the most probable contacts a user will call at a given time and displays them for the user. Several solutions have been proposed in this category [3] [6] [8] [24] [25] [26] [27]. One of the most relevant algorithm was proposed by Plessas et al. [6] [8] which uses recency and frequency of communication of contacts as input and achieves an average prediction accuracy of 80%. We will discuss this algorithm in more detail in the section below.

One problem that is faced in call prediction based approach, is deciding the level of granularity at which one should analyse the call data. Sarkar et al. [28] proposed a time-series segmentation technique to extract optimal time segments of similar user behaviour. They first generate some initial time slices by dividing the data into a small base period. Then, behaviour-oriented segments were generated by iteratively finding the dominant behaviour of each time slice and aggregating it with adjacent slices with the same dominant behaviour. We note here that a user’s context is constantly changing along with his/her phone usage and hence recent data is more useful for predicting the users future behaviour. This motivated Sarker et al. [29] to design an algorithm in which the input are complete smartphone logs and the output is the optimal time period in which the users behaviour is similar to the current behaviour. In the final step of the algorithm, new rules are derived from phone logs for this time period and outdated rules are removed.

### The FR algorithm

In this section, we will discuss in detail two relevant papers on predicting future outgoing calls. Stefanis et al. proposed an algorithm to predict future outgoing calls using two contextual indicators: Frequency and Recency [8]. They first performed several experiments to prove that these two dimensions play an important role in predicting future outgoing calls. They used Nokia MDC Dataset for their experiments [10]. They divided the users in to three major groups: the group of least social users, group of average social users, and the group of most social users. They used cross-validation technique to evaluate the FR algorithm. They argue that users call logs may be redundant after specific lengths and hence introduced a training window. The calls in the training window are used as training data for prediction of future outgoing calls. The length of training window may vary depending upon the level of *socialness* of a user, but the time span of the training window remains fixed for all users. They found that the algorithm performed its best when the length of the training window is between 10 to 15 days and recency window is between 6 to 12 hours.

They calculated the prediction score of each contact by summing up the weighted frequency and recency scores. The score for a contact *c* is calculated using the following formula:

$$\begin{array}{c} \Pi \left(c\right)=w_{f}\times F\left(c\right)+w_{r}\times R\left(c\right) \end{array}$$

Here, Π(*c*) is the score computed for contact *c*. While *w*_*f*_ is the weight of the frequency score *F*(*c*) and *w*_*r*_ is the weight for the recency score *R*(*c*). The weights assigned to the frequency and recency score depend upon the socialness of a user and size of prediction list. We note that *F*(*c*) is the percentage of the communications between the user and the contact during the training window. While *R*(*c*) is a percentage of the time interval between the start of a defined recency time window until the most recent communication between the user and the contact.

Once the prediction score for each contact is calculated, the top contacts are shown to the user as the most likely contacts that she will call. The number of contacts shown to the user depends on the screen size of the smartphone. Stefanis et al. evaluated their algorithm on a prediction list of sizes: 1, 3, 5, and 8 contacts. Their algorithm was successful 90–95% times for the least social group, 78–86% times for the average social group, and for the most social user group, the accuracy was 69–73%.

In an extension of this work, Plessas et al. performed a field evaluation where they evaluated the performance of the proposed algorithm [6]. They incorporated their prediction algorithm in an Android application and asked their participants to use it for 30 days. They found that on average 59% of 103 participants made their calls using their application and almost 78% times the application suggested a list that included the contact they intended to call.

## Limitations of FR algorithm

In this section, we will discuss some of the limitations of the FR algorithm [8] [6]. We observed that the FR algorithms can be improved in two aspects. We will discuss them one by one below. We used the smartphone dataset [3] because other than being more recent it is collected from a larger set of users. We recall that after filtering, we were left with 522 users in the smartphone data set and 129 users in the MDC dataset.

### Performance on top contacts

We have seen earlier that humans are biologically designed to maintain close social contacts with nearly 3–5 people [30], and this is reflected in mobile phone communications as well [30]. Hence, we computed the average probability of communication with top 3 contacts which turned out to be 0.41 for the smartphone dataset. Here, top contacts of a user are most frequently called contacts. We calculated them by analyzing the dataset in R. We are predicting future outgoing calls based on the historical calling patterns of a user.

**Fig 1:**
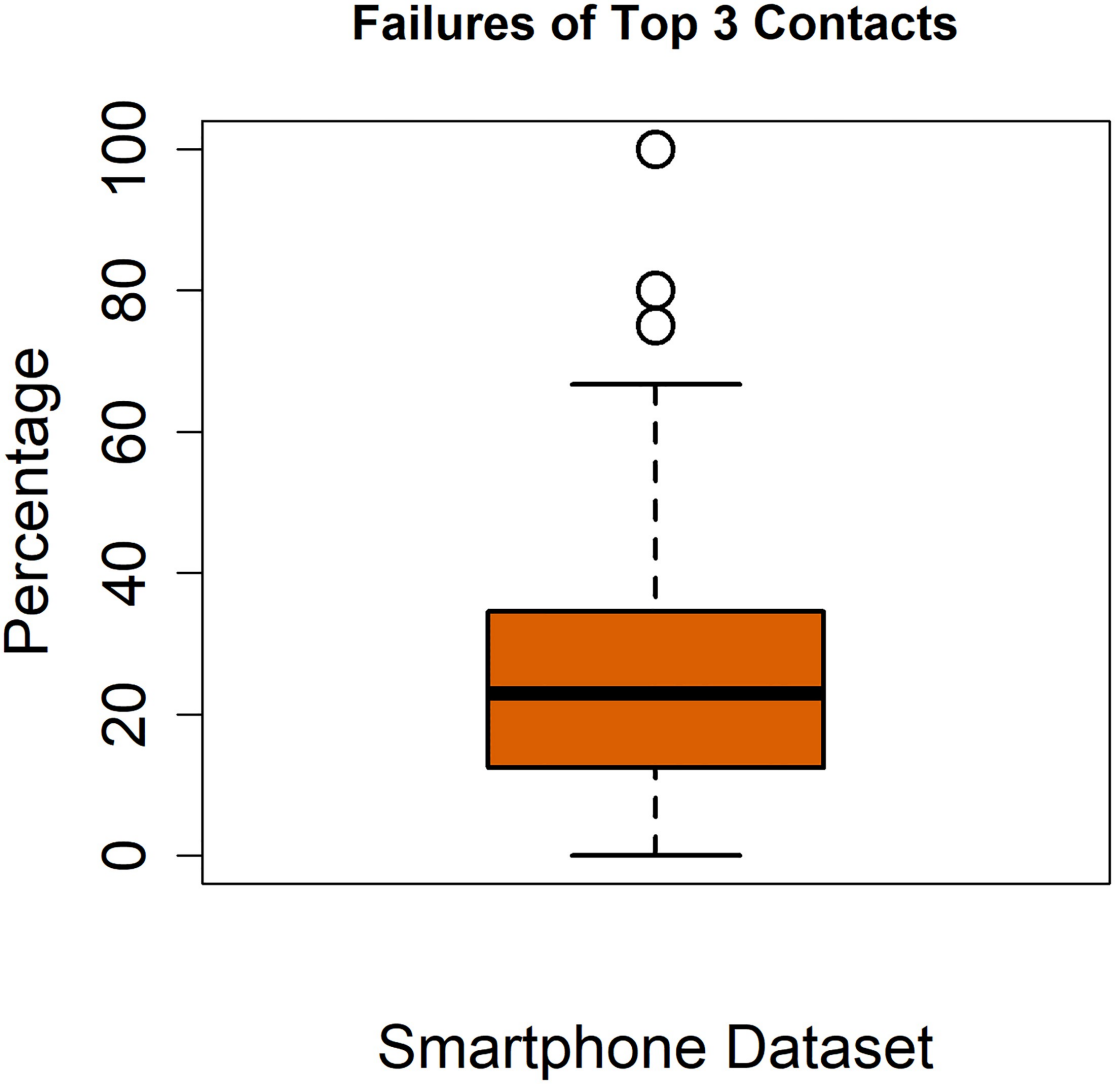
Failure of top 3 contacts. Boxplot showing the number of times prediction failed for the top 3 contacts.

We then wanted to test how the FR algorithm performs in terms of its performance on correctly predicting a contact in the top 3 contacts of a user. This was done by implementing the FR algorithm in R and simulating it on the smartphone dataset. We divided the data of each user’s call log into two sets. First set comprises of 80% of the data and the other half has 20% of data. The simulation was done by first training the dataset on 80% of the call data for each user and then testing it on the remaining 20% of the data. We used sliding window similar to [3] for training set for this purpose. After each iteration, the tested call is added into training set and one most recent call from training set is shifted to the testing set. We found that on average the algorithm failed to predict a call to one of the top 3 contacts 26.83% times (s.d 22.087) as shown in Fig 1. This is rather high. We suspected the reason might be that the FR algorithm assigns high recency and high frequency score to the same contacts. This is problematic as these scores captures two different aspects of the importance of a contact; and if there is a big overlap, then the benefit gained is reduced. To test our hypothesis, we computed the overlap between the *k* contacts with the highest recency scores and the *k* contacts with the highest frequency score. We computed the common contacts between these two sets for different values of *k*, i.e. 1, 5, 10 and 15. Table 1 shows that for the different length of lists there is a high percentage of common contacts. This is because the frequency score is computed from the call-log in a time window of limited duration. Thus, it is possible that the contacts with top *k* recency score and the top *k* frequency have a large overlap due to increased interaction due to a social event (e.g. dinner) or professional reason (e.g. project deadline).

**Table 1:** Common contacts between the most recent and frequent contact lists.

	*k* = 1	*k* = 5	*k* = 10	*k* = 15
**Smartphone Dataset**	34.01%	50.94%	59.82%	73.52%

Table showing the percentage of common contacts between the most recent and frequent contact lists for different lengths *k* of prediction lists. It shows that for different lengths of lists there is a high percentage of common contacts.

### Temporal patterns in call logs

Human life is organized in recurrent patterns of activities. Some of these recurrent patterns are due to natural reasons such as circadian rhythms which determine the time we sleep or eat. However, other recurrent patterns have no biological basis and determined based upon various social conventions and are known as *socio-temporal patterns*. For example, sociologists argue that various social events such as visits, and exchanging letters have a fairly uniform temporal spacing [31]. Hence, it is no surprise that various studies have investigated the effect of these recurrent patterns on the calling behaviour of users, including circadian rhythms [31] and seasonal variations [32]. Similarly, the study of Nasim et al. [3] indicates that various socio-temporal patterns exist in the calling behaviour of users and they can be used for predicting calls.

**Fig 2:**
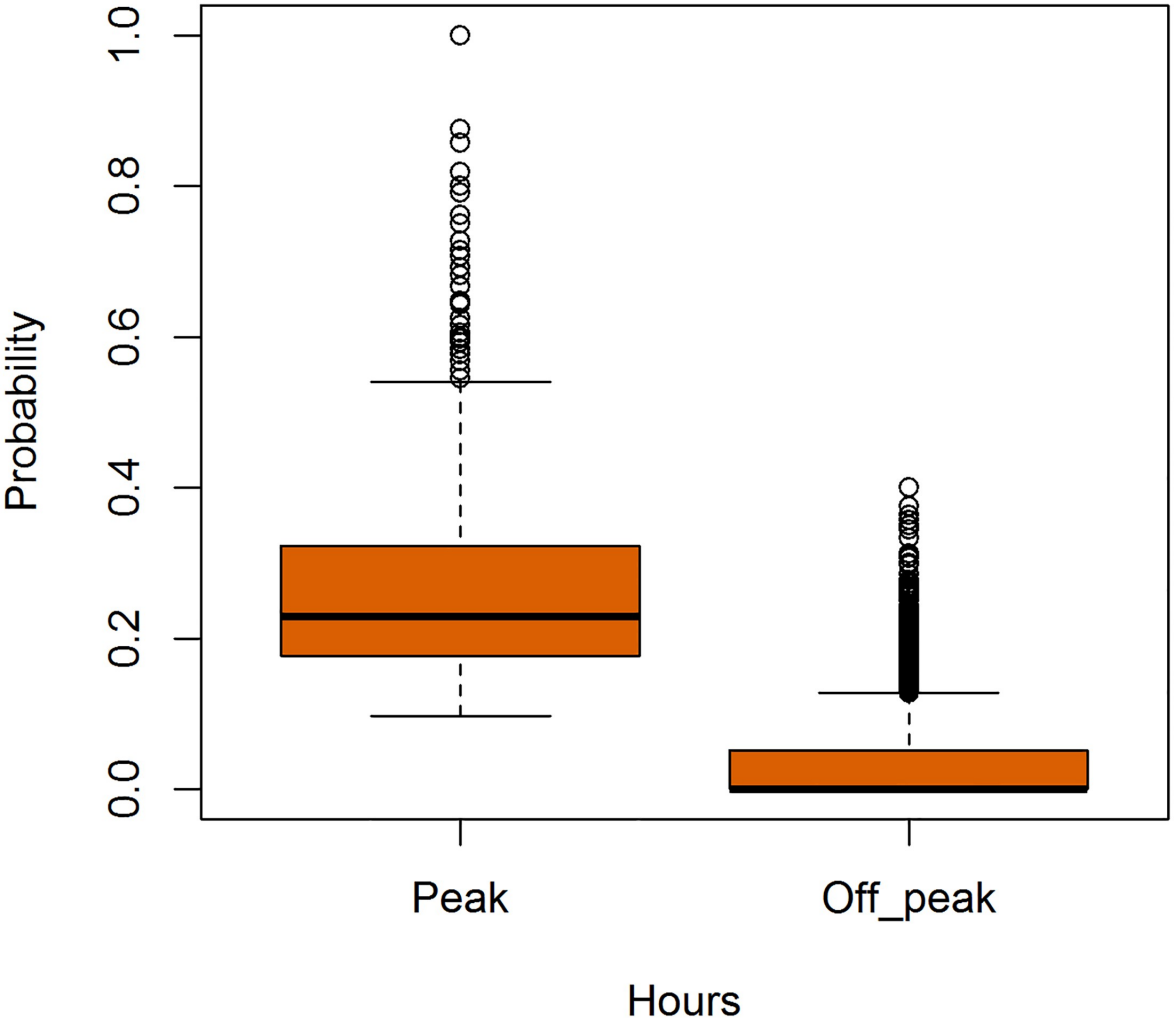
Probability of calls in specific hours. We can see that more communication happens at the peak hour.

**Fig 3:**
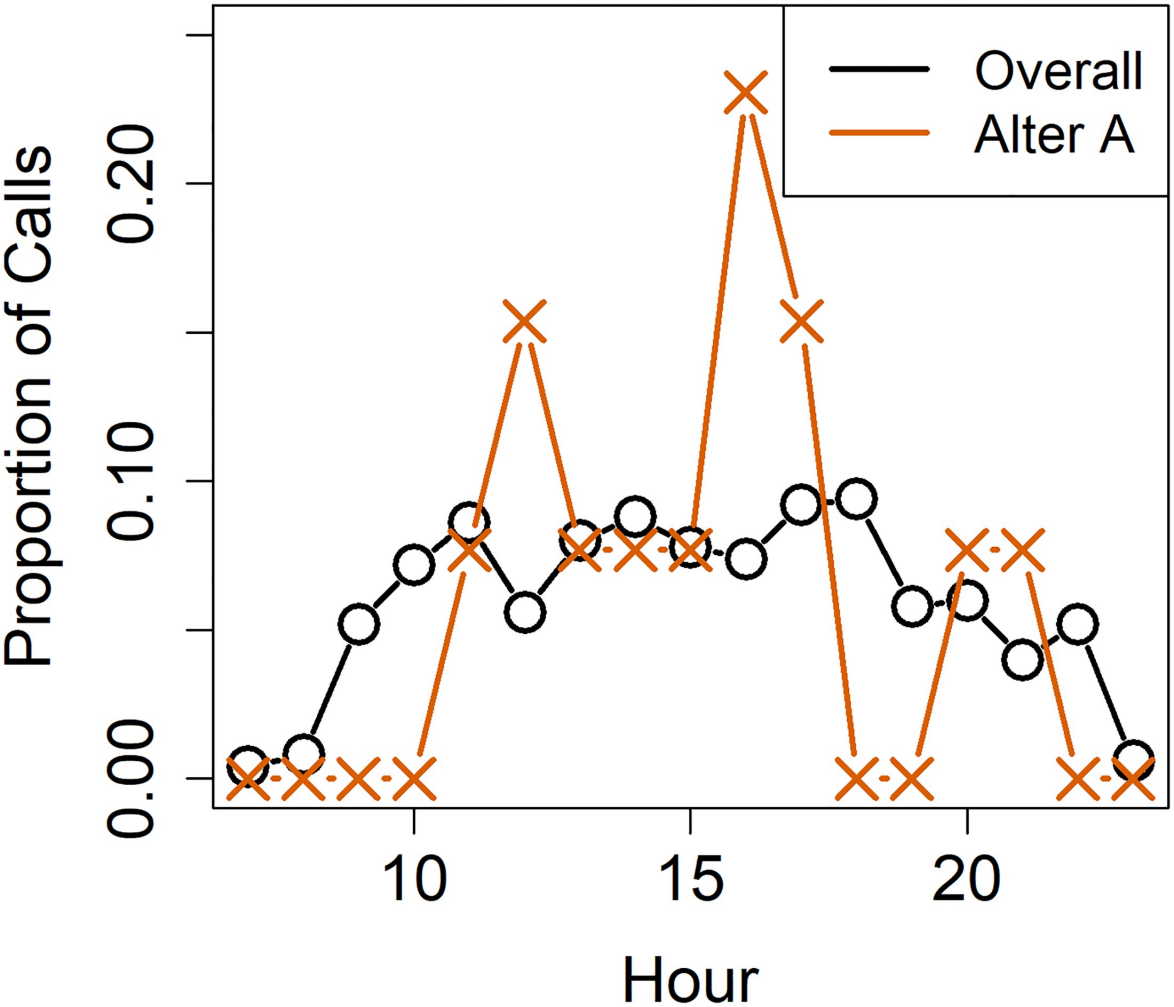
A figure showing that although the calling frequency is almost consistent during the waking hours of the user, however he/she tends to call one particular contact more during particular hours of the day. Specifically, the calling frequency is significantly higher at one particular hour called the *peak-hour*.

We extracted one such pattern by noting the hour of the day at which a call is made and then computing the probability of an user calling a particular contact for each hour of the day. Interestingly, we found that there is a preferred hour for contact between a user-contact pair that we call as *peak hour*, the remaining hours of the day are denoted as *off peak hours*. This behaviour can be attributed to various social reasons. For instance, for a professional user-contact setting, a reason like the user calls a contact for a meeting around 9:00 am/pm every few days seems plausible. We can see from Fig 2 that the distribution of probability of calls between each user-contact pair in our dataset. An example of peak hours can be seen in Fig 3 which shows that although the calling frequency is almost consistent during the waking hours of the user, however, she tends to call one particular contact during specific hours of the day called the peak-hour.

We then computed the mean probability of calls for a pair at the peak hour as (*μ*_0_) and the mean probability of calls for each pair at the off peak hours (*μ*′). We calculated *μ*_0_ to be 0.22 and *μ*′ to be 0.03. We then applied hypothesis testing on the following set of hypothesis:

$$\begin{array}{c} H_{0}:\mu_{0}=\mu^{\prime } \end{array}$$

$$\begin{array}{c} H_{A}:\mu_{0}>\mu^{\prime } \end{array}$$

We found that this difference is statistically significant with a p-value of 2.2 × 10^−16^ at a significance level of 0.95.

The next section describes the improved version of FR algorithm, which incorporates periodicity as a third dimension for improving call prediction task.

## Proposed algorithm

In the last section, we saw two limitations in the FR algorithm. The first limitation is that the FR algorithm fails to predict a contact in the *top-3* because it computes frequent contacts in a time window. We instead propose that we should compute both the overall frequent contacts, but also the contacts that were frequently contacted recently. Secondly, the FR algorithm does not incorporate temporal patterns in terms of peak hours, which we can overcome by incorporating them.

Hence, the proposed algorithm considers three parameters: recency, frequency, and periodicity, to calculate *contact Score* *S*_*u*_(*c*) for user *u* and her contact *c*. The contact score is given by:

$$\begin{array}{c} S_{u}\left(c\right)=R_{u}\left(c\right)+F_{u}\left(c\right)+P_{u}\left(c\right) \end{array}$$

Here, *R*_*u*_(*c*) is the recency score, *F*_*u*_(*c*) is the frequency score and *P*_*u*_(*c*) is the periodicity score for each user and each contact of her. The algorithm computes this score for each contact and then recommends the top *k* contacts as the most likely contacts the user will call at a given time *t*. The value of the variable *k* is decided on parameters such as how many contacts can be shown given the screen size of the phone. We note here that an average mobile phone can easily show 5–7 contacts to a user at a time. A brief description of how these scores are calculated is given below:

**Recency Score** is computed for the contact *c* by measuring the time elapsed between the current time and the last contact between *u* and *c*. We split the time in to four time windows and assign a lower score as we move further back in time. Let Δ*t* represents the absolute time difference between the user’s attempt to make a call and the time the contact was last contacted. Then, the recency score assigned to each contact can be represented by the following expression:
  $$\begin{array}{c} R_{u}\left(c\right)=\{\begin{array}{cc} 5-\lfloor \frac{\Delta t}{24}\rfloor & \;\text{if}\;0\leq \Delta t\leq 120\\ 4 & \;\text{if}\;24\leq t_{c}<48 \end{array} \end{array}$$
  All contacts who were contacted during the last 24 hours are assigned a score of 5, contacts contacted in last 48 hours are given a score of 4 and so on.
**Frequency Score** is an aggregate of two sub-scores for each contact: the overall frequency *F*_*o*_(*c*) and recent frequency *F*_*r*_(*c*) scores. A higher overall frequency score is assigned based on the whole call-log, while the recent frequency score is assigned based on the data of the last 12 hours. We check if an contact lies in the top 3, top 7 or top 15 most contacted contacts both recently as well as in the overall case. These grouping were decided by keeping in mind Dunbar’s number [30]. These groups are then assigned scores as given in Table 2:
  The net frequency score is then computed by the following:
  $$\begin{array}{c} F_{u}\left(c\right)=F_{r}\left(c\right)+F_{o}\left(c\right) \end{array}$$
**Periodicity Score:** is calculated by using *peak hours* of each user-contact pair as discussed in Section. It is calculated by analysing the closeness of a specific contact’s *peak hour* with the current time. Here, the current time is the time for which we are trying to predict the next call. If the peak hour lies within ±2 hours of the current time, then we assign a score of 7, if its within ±5 hours then we give it a score of 4, if its within ±6 hours then the assigned score is 3, if its within ±8 hours then the assigned score is 2. We give a periodicity score of 0 otherwise.

**Table 2:** Scores assigned to frequent contacts.

contact Group	Top 3	Top 7	Top 15
Frequency Score	7	5	3

In the next section we present the result of our evaluation of our proposed algorithm.

## Evaluation and results

We implemented our proposed algorithm in R to evaluate its effectiveness in predicting calls. We also implemented the algorithm by Stefanis’s [8] for comparison. We performed analysis on the two previously mentioned datasets: Nokia MDC dataset [10] and smartphone dataset [3].

We adopted cross validation technique to validate the effectiveness of our proposed algorithm. The evaluation was designed to simulate a user’s real life experience of making a call with a smartphone that runs our proposed prediction algorithm. When a user opens a dialler she will be presented a *prediction list* of *n* contacts. The prediction will be *successful* if the user selects an contact to show her intention of calling, and it will be *unsuccessful* if the user actually makes a call to another contact. Smartphones have smaller sized displays and hence limited number of contacts can be shown without the need of scrolling. Since usability will decrease if a user scrolls, so we limit of prediction list size to sizes that can be easily displayed. A typical screen on a smartphone can show between 5–10, hence, we evaluate our algorithm on these prediction list sizes.

**Fig 4:**
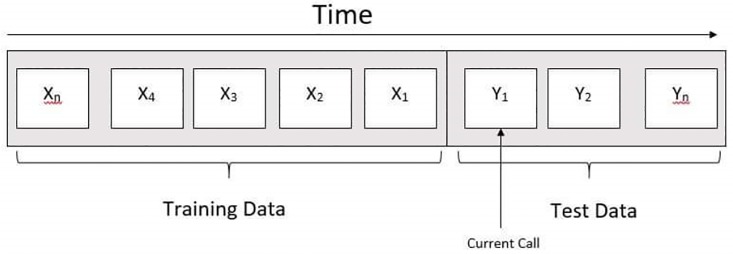
Division of user’s call log using cross validation technique.

Our evaluation algorithm first divides our data into a training set containing 80% calls of a particular user and a test set containing the latest 20% calls. Here, we used the same approach as used by Nasim et al. [3] This division is diagrammatically described in Fig 4. We pick the call *Y*_1_ in the test data and note its time. We then compute the score of each contact in the training data, as discussed in Section. We then form the prediction list from the *k* contacts with the highest score. Once a prediction list is generated, we consider the prediction a success if the contact of *Y*_1_ is in the list, otherwise, we consider it a failure. The call *Y*_1_ is then made part of the training data and the process is repeated for the next call in the test data. Once all the calls in the test data are exhausted, we calculated the overall accuracy for the entire data. Note that accuracy is the primary parameter used to evaluate call prediction algorithm by previous studies [3] [8]. Accuracy is calculated by using the following equation:

$$\begin{array}{c} Accuracy=\frac{total\;success\;count}{total\;success\;count+\;total\;failure\;count} \end{array}$$

### Overall performance

We calculated the accuracy of the algorithm for different sizes of prediction lists to check the effectiveness of our algorithm for different list sizes. In Table 3, we presented the accuracy of both algorithms for different sizes prediction lists (5, 7 and 15). We selected different sizes of prediction lists according to screen sizes of modern smart phones. We can clearly see from Table 3 that the accuracy is higher for larger sizes of the prediction. We however add that a large list size is less useful to the user. Hence, we suggest that a list of 7 would be optimal in terms of the trade-off between containing the needed contact and need of scrolling with a larger list size like 15 on some screen sizes.

**Table 3:** Performance of the two algorithms for all contacts. This table presents the accuracy for all contacts. We show results for prediction list sizes of 3, 7 and 15.

List Size	MDC Dataset		Smartphone Dataset	
	Proposed Algorithm	FR Algorithm	Proposed Algorithm	FR Algorithm
5	0.80	0.77	0.44	0.44
7	0.85	0.79	0.56	0.51
15	0.92	0.88	0.58	0.56

### Analysis for top contacts

We now analyse how our proposed algorithm performs in terms of top contacts. For this analysis, we first extracted those calls that were initiated to one of the top *k* contacts from the test data, and then checked how they performed in terms of accuracy, i.e., what percentage of calls to the top contacts were correctly predicted.

The result of our analysis is summarized in Table 4. In Table 4, we compared the accuracy (performance) of algorithm for top contacts (3, 7 and 15). It represents that how accurately our algorithm work for top contacts. It shows that our proposed algorithm outperforms the FR algorithm for both datasets. Moreover, this difference is more predominant for the smartphone dataset. We recall that earlier in the paper, we showed that the smartphone dataset more accurately represents the modern mobile phone users as the MDC dataset was gathered a number of years ago. Hence, we can conclude that overall our proposed algorithm will correctly predict the contact if it is one of the top contacts of the user.

**Table 4:** Performance of the two algorithms for top contacts only. This table presents the accuracy of algorithm for top contacts. We show results for prediction list sizes of 3, 7 and 15.

Num Contacts	MDC Dataset		Smartphone Dataset	
	Proposed Algorithm	FR Algorithm	Proposed Algorithm	FR Algorithm
Top 3	0.99	0.96	0.94	0.86
Top 7	0.94	0.90	0.87	0.78
Top 15	0.88	0.84	0.74	0.71

### Comparison of algorithm for different socialness level

Stefanis et al. [8] compared the performance of their algorithm for users with different socialness level. They calculated the socialness of a user *u* by first generating percentage of commuinication to different contacts, i.e the proportion of communication *f*_*i*_ devoted to each contact *a*_*i*_ of an user < *a*_1_, *f*_1_ >, < *a*_2_, *f*_2_ >, ⋯, < *a*_*n*_, *f*_*n*_ > such that *f*_*i*_ ≥ *f*_*j*_ when *i* < *j*. The socialness level *s*(*u*) of that user is then calculated using the following formula:

$$\begin{array}{c} s\left(u\right)=f_{1}+\sum_{i=2}^{n}\left(f_{i}-f_{i+1}\right) \end{array}$$

Stefanis argues that a lower *s*(*u*) shows that the user communicated with more contacts with similar proportions and hence is more social than a user whose with a higher socialness score.

**Fig 5:**
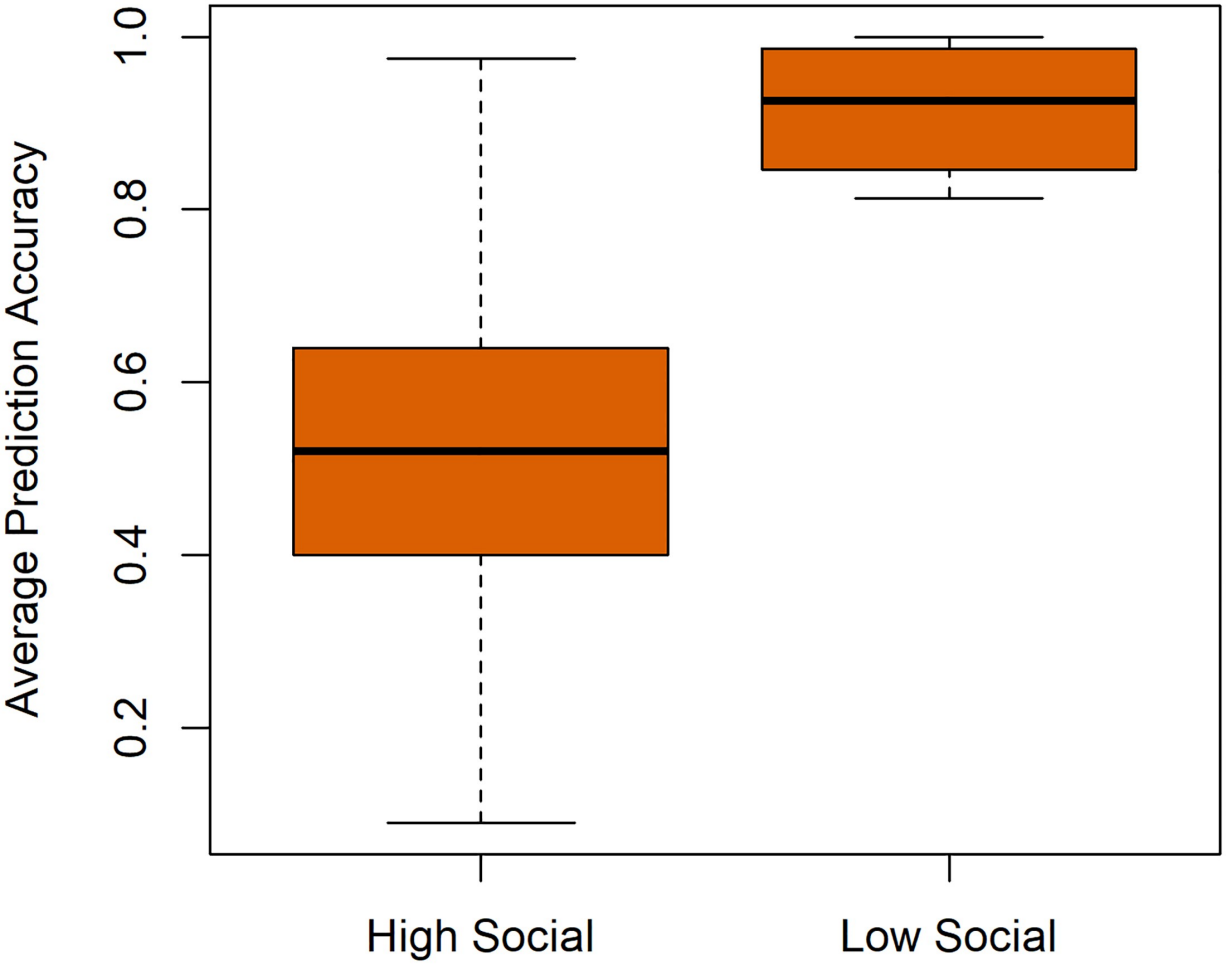
This figure shows how our algorithm performs for users with different socialness level. We can see that the algorithm is more accurate for low social users.

After calculating the socialness score of each user, we divided our 522 users left after data cleaning in the smartphone dataset into two groups using k-mean clustering algorithm. Note for this analysis we only used the smartphone dataset as we have seen earlier that it better represents present day users. We then checked the accuracy for these two groups. We used a prediction list size of 5 only as it is more difficult to correctly predict a contact when the list is shorter. Fig 5 shows a boxplot summarizing the performance of our algorithm for both user groups. We can see that the algorithm is more accurate for low social users. The possible reason can be that most of their communication is directed to a very small subset of contacts.

## Conclusion

This paper enhances the effectiveness of the FR algorithm for call prediction [8]. This algorithm uses the frequency and recency of a user’s contacts for predicting which contact a user will call at a given time. We first show that the FR algorithm can be enhanced in two aspects. The first is that its performance in predicting top contacts is not to the satisfaction of users. Secondly, user’s calling behaviour contains peak-hours for each contact which can be used as an additional contextual cue for call prediction. The top contacts were missed because the standard FR algorithm uses a time window to calculate the *k* frequent contacts which occasionally misses the most important contacts of a user. These issues were then addressed in the following manner:

1. The issue of missed top 3 contacts was addressed by generating the list of *k* most frequent contacts both in a time window and in the overall data of the user.
2. The FR algorithm was further enhanced by adding temporal information about peak-hours of contacts as an additional dimension.

Our simulation showed that the proposed algorithm is more effective than the standard FR algorithm for both datasets and various prediction-list sizes. Similarly, the prediction accuracy for the top 3 contacts also improved in the proposed algorithm. However, like all research studies, this paper also has its limitations. The main limitation is that the dataset is from a particular user group and hence its results cannot be generalized. Hence, one possible future research direction is to collect data from different user groups and see if they have similar communication behavior, and also assess the algorithm’s performance. Moreover, it is not known how users will react to such a prediction based calling interface in practice. This will require a more formal *in the wild*, usability study.
